# Cancer information on the Internet

**DOI:** 10.3332/ecancer.2007.ed3

**Published:** 2007-10-30

**Authors:** Gordon McVie

**Affiliations:** European Institute of Oncology, Milan, Italy

It is the perpetual perception of having too little information which drives cancer patients to search the Internet. This in turn creates concerns for doctors, not only that the Internet will harm their patients, but that it might cost more time in consultation, thus lose them income, and, perish the thought, perhaps challenge their authority and reputations. Many clinicians’ have a biased view that only information handed down by themselves will be helpful to patients. This like many other such beliefs, is not evidence-based [1]. Trying to find that evidence, however, is a thankless task, as there is not much in the conventional peer reviewed literature. What there is suggests a total lack of negative effects of hunting cancer information on the web [2]. But is this topic appropriate for peer review in any case? And who are the peers? Surely other patients are the peers, and the process might be re-christened “peer view”.

There is considerable evidence, particularly from the sheer numbers of self-help web-based communities in cancer, and their durability, that patients willingly trade “views” and cancer information, and accurately, too [3]. A recent study of 4600 consecutive postings of information during a three month period on Breast Cancer Mailing List showed ten errors (0.22%). Seven of these were corrected by other readers of the website within nine hours, average of four hours [4]. Compare this to the average error rate, (mostly omissions) in a weighty paper-based oncology textbook which takes three years to compile and several hundred dollars to purchase. This year’s ASCO announced 74 new therapeutic molecules, none of which will have been mentioned in the latest cancer tome. The end is already in sight for paper as an information medium, according to Bill Gates, but he would say that wouldn’t he?

Though the pace is being set by the younger generation, the older brigades who get most of the cancers are catching up! Take a look at Web2.0 [5]. The next generation of the web. This is where the web has evolved and improved over time and now offers better and more up to date services like blogs, wiki’s and social networking sites. This is bottom up information run by, and for consumers, be they using book reviews, hotel star ratings or doing their banking or shopping online. Most clinicians are not involved in the new interactive, online world.. How many of us believe the Nature article which claimed that information about science was not greatly inferior on Wikipedia (written mostly by amateurs) compared to Encyclopedia Brittanica? [6] The former is classic Web2.0 , while the latter is conservative, “quot;doctor knows best” Web1!

Wikipedia has come from nowhere to contain over three and a half million entries in two hundred and twenty languages in a decade. Small wonder that it attracts cancer patients, who are instinctively suspicious of IT contraptions such as that being instigated by Britain’s National Health Service. This is a ludicrously expensive system which is the control freak’s ultimate dream- central command and control, designed by managers for managers (and their political masters). Will such a system help inform the cancer patient about their medication, its side effects and the management of the said effects? Not a chance. If it delivers an appointment or a parking space at the hospital, it will be amazing; if it provides email access to the patient’s physician it will be a miracle! No, the patients have already voted with their fingers or Voice Control systems, on laptops, multimedia tv sets, palm tops or cellphones. And this is not the exclusive domain of the worried wealthy, but is increasingly possible in developing countries, where language solutions are being found (see Wikipedia, above), and new media options are dropping in cost.

It would have been reassuring for oncologists to have had secure cancer information systems in place ahead of the Web explosion, or at least a rating for trustworthy blue chip academic websites, but the opportunity has been missed. So we have no option but to trust our patients to find relevant information for themselves, although meantime we could do worse than get up to date on the technology, and point them in the right direction.
